# Effects of Horseback Riding on the Postural Control of Autistic Children: A Multiple Baseline Across-subjects Design

**DOI:** 10.1007/s10803-023-06174-5

**Published:** 2024-01-21

**Authors:** Juan Vives-Vilarroig, Paola Ruiz-Bernardo, Andrés García-Gómez

**Affiliations:** 1 Universidad Jaume I. Castellón, Av. Vicent Sos Baynat, s/n, Castellón de la Plana, 12071 España; 2 Universidad de Extremadura. Badajoz, Av. de Elvas, s/n, Badajoz, 06006 España; 3 Universidad Cardenal Herrera, CEU, Castellón, C. Grecia, 31, Castellón de la Plana, 12006 España

**Keywords:** Horse-assisted activities, Autism spectrum disorder, Postural control, Horseback riding therapeutics

## Abstract

The aim of this research was to study the effect of a horseback-riding programme on postural control in a group of autistic children (ASD). Nine children aged 9 to 12 years participated in this study through a multiple baseline across subjects design. The whole programme took place over nine months. Participants followed a previously developed specific horseback-riding programme, consisting of 45-minute sessions held twice a week for at least three months. To evaluate postural control, the average velocity of the centre of pressure displacement was measured by means of a posturographic platform. Results indicated that this intervention with horses had a positive effect on the postural control in children with ASDs.

## Introduction

Autism spectrum disorder (ASD) is a neurodevelopmental disorder characterised by a significant deficit in social communication and repetitive behaviours, restricted interests, and/or atypical sensory behaviour. The spectrum of autism refers to the great variability of the symptoms presented by each affected individual. ASD is often associated with other disorders or medical conditions and may co-occur with intellectual disability and language impairment, ranging from mutism to articulatory and grammatical correction. In all cases adaptive skills are significantly impaired and can be classified into three levels of severity depending on the support required (APA, 2013).

In recent years, ASD has become one of the most prevalent neurodevelopmental disorders in children due, partly, to new diagnostic criteria, greater sensitivity in society, and increased specialist training (Hyman et al., 2020). It is currently estimated that one in 59 children under the age of 8 years has symptoms that meet ASD criteria (Maenner et al., 2020).

### ASD Associated Disorders

ASD is a complex reality and has often been associated with other disorders such as stress (Ogawa et al., 2017; Corbett et al., 2016), anxiety (Ezell et al., 2019), sleep (Neumeyer et al., 2019), gastrointestinal (Madra et al., 2020), obsessive–compulsive (Martin et al., 2020), attention deficit hyperactivity (Muskens et al., 2017), sensory processing (Rydzewska et al., 2019), and motor disorders (Licari et al., 2020), among others. There is often a circular relationship between some of these disorders and the expression of ASD symptoms. For instance, greater sleep disturbances may induce higher severity of expression of ASD symptoms and vice versa, as do anxiety, sensory processing disorders, and motor disorders (Cohen et al., 2014; Mazurek & Petroski, 2015).

The relationship between some of these disorders and ASD is sometimes so close, as in the case of motor disorders, that some authors have even considered them to be typical of autism (Crespo-Eguílaz & Narbona-García, 2009; MacDonald et al., 2012, 2013).

### Motor Disorders and Postural Control

Although not regarded as an essential symptom of ASD, motor disorders appear as an associated symptomatology in the Diagnostic and Statistical Manual of Mental Disorders Fifth Edition (APA, 2013), as these symptoms seem to show a peculiar and different functioning from other conditions such as developmental coordination disorders. (Caeyenberghs et al., 2016; Valverde-Esteve et al., 2020). Dziuk et al. (2007) have suggested that movement impairments in ASD have more similarities than differences with impairments found in developmental coordination disorder. Jansiewicz et al. (2006) found impaired balance and gait, slower speed of timed movements and greater “sprawl” movements in autistic children than in normotypical children. However, the breadth of the autistic spectrum makes it difficult to establish a common pattern.

Also, Fournier et al. (2010) argued that the deficit of postural control, which is basic for a good balance control, is an aspect that is repeated throughout the autism spectrum, presenting a less stable and more variable posture in comparison with neurotypical children of the same age. This was also endorsed by Lourenço et al. (2020) since, in his research, autistic children presented significantly lower scores in relation to motor skills, including balance. In several studies, such as that of Fernándes (2020), it was observed that autistic people scored significantly lower on the balance scales. Similarly, Larson and Mostofosky (2009) found in their work that autistic children have greater difficulty in balance and gait compared to normotypical children.

Moreover, motor disorders can help to establish the diagnosis and obtain prognostic indicators regarding the adaptive behaviour of these individuals (Stevenson et al., 2017). However, although very common, motor disorders are often not systematically assessed by diagnostic teams (Licari et al., 2020).

The typology of motor disorders in ASD is very diverse. Some of the most common problems are difficulties in general dynamic coordination, problems in flexibility and motor tone, gait disorders, difficulties in fine motor coordination, motor praxis, and difficulties in postural control and balance (Paquet et al., 2016; Wang et al., 2016; Molloy et al., 2003; Lum et al., 2020). Among these motor difficulties, balance disorders are of particular interest as they directly affect the success of carrying out everyday activities (Travers et al., 2017). Balance is the ability of our body to remain upright through compensatory movements when stationary or in motion (Mosston & Ashworth, 2008). Balance provides the body with a stable basis for locomotion and manual and facial actions, thus facilitating effective interaction with the environment, both physical and social (Adolph & Franchak, 2017). In fact, everyday tasks such as soaping and getting out of the shower, cooking, or dressing become more difficult if there is a lack of good stability in the upright position (Smith & Fisher, 2018). We use the term ‘postural control’ to refer to the complex ability that involves different sensory–motor processes and that aims to achieve a correct balance in both static and dynamic activities (Winter et al., 1990; Westcott et al., 1997). Guzmán-Muñoz et al. (2019) consider that good postural control is necessary for correct balance in both static and dynamic activities. Montes-Castillo et al. (2000) believe that postural control helps the body to be stable and stay balanced.

Balance is a complex behaviour in which three sensory systems act in a coordinated way: the vestibular system, which determines the position of the head with gravity and the movements of acceleration and deceleration of the head (Ayres, 2008); vision, which provides information on the position of the body in relation to space and objects (Aartolahti et al., 2013; Bronstein, 2019); and the proprioceptive system, which intervenes in the processes of discrimination and localisation of body parts, helping to modulate the force of contraction, temporalisation of movement, and straightening reactions, among others (Speers et al., 2002). When these three systems function correctly, balance issues are usually absent. However, when one of these systems does not function correctly, due to neurophysiological alterations (Maurer & Damasio, 2008; Nayate et al., 2005), compensatory mechanisms involving the remaining sensory systems may make postural control possible, though less effective (Lakie & Loram, 2006; Parreira et al., 2017).

### Balance Improvement Interventions

Due to the important role of balance in a person’s adaptive capacities, it is essential to develop interventions to improve balance in children in whom this skill is impaired (Wuang et al., 2020; Stins & Emck, 2018; Peters & Wood, 2017).

Programmes that stimulate the sensory systems and aim to improve balance in people with acquired brain damage or cognitive impairment have been successful, but interventions aimed at improving balance in autistic children are very rare and have yet to yield definitive results (Hilton et al., 2014; Dobell et al., 2020). Although results are not conclusive, they have been encouraging with sensory stimulation interventions (Sam et al., 2017), visual feedback through video games (Travers et al., 2018; Hilton et al., 2014; Somogyi et al., 2016; Peña et al., 2020), gymnastic and neuromuscular training (Akyol & Pektas, 2018; Najafabadi et al., 2018; Shavikloo & Norasteh, 2018), therapeutic skating (Casey et al., 2015), Tai Chi Chuan (Sarabzadeh et al., 2019), and also with the help of elephants (Nuntanee & Daranee, 2019) and horses (Portela-Pino et al., 2019).

Hariri et al. (2022), in a systematic review of the literature, shed light on the available intervention strategies for improving postural control in people with ASD. This study has reported that most interventions affect motor function in ASD by improving cerebellar or balance function, integration and processing of sensory information, muscle strength and correction of body alignment. Nevertheless, all these studies have limitations associated with insufficient sample size and the lack of a control group which could potentially compromise the validity of the results of such studies. This situation was also observed in a systematic review by Trzmiel et al. (2019) where specifically equine-assisted activities and therapies in autistic children were studied. It was concluded that, although the different studies claim an effective impact of the therapies, as previously shown in the previous literature review, the available publications often present flaws in the methodology, mainly derived from the small sample size and the lack of a control group. The use of different therapeutic protocols, and especially different methods to measure efficacy, often reduce the quality of a study.

### Horseback Riding Benefits

Horse-riding has a high potential to facilitate the postural control of autistic children and thus improve their balance, since it is an activity in which multiple stimuli from different sensory channels need to be coordinated (Hameury et al., 2010). It is also a highly specific intensive exercise for improving balance, since the horse’s gait induces continuous changes in the rider’s centre of gravity requiring immediate positional readjustments to avoid falling off (Bronson et al., 2010; Muñoz Lasa et al., 2015; Tseng et al., 2013). Moreover, horseback riding meets two other criteria related to the successful care of autistic people: it is a motivating and highly structured activity (Kern et al., 2011; García-Gómez et al., 2014).

In recent years, multiple studies have emerged on the benefits of horseback riding, especially for people with ASD. Peters and Wood (2017) conducted a systematic review of the scientific literature on the topic, focusing on studies with interventions. The authors concluded that most of the studies had methodological weaknesses, but collectively provided evidence that people with ASD who participated in these interventions experienced improvements in social interaction, positive emotions, stress, communication, and especially motor skills. An important finding of this study highlights some authors’ proposal that the sensory nature of riding, including gradual vestibular, proprioceptive, and tactile input, promotes self-regulation in children with ASD, which, according to Gabriels et al. (2012), contributes to a feeling of calm in a child with ASD. Such evidence is consistent with the scientific literature that suggests interventions that include graded sensory input for children with ASD can improve academic responsive behaviour, concentration behaviour, and stereotypical behaviour (Jenkins & Reed, 2013; Bass et al., 2009).

### Objective

The aim of this research was to study whether a therapeutic programme based on horseback riding can improve postural control in autistic children, as measured by postural control data from standing on a platform.

## Materials and Methods

### Study Design

**Fig. 1:**
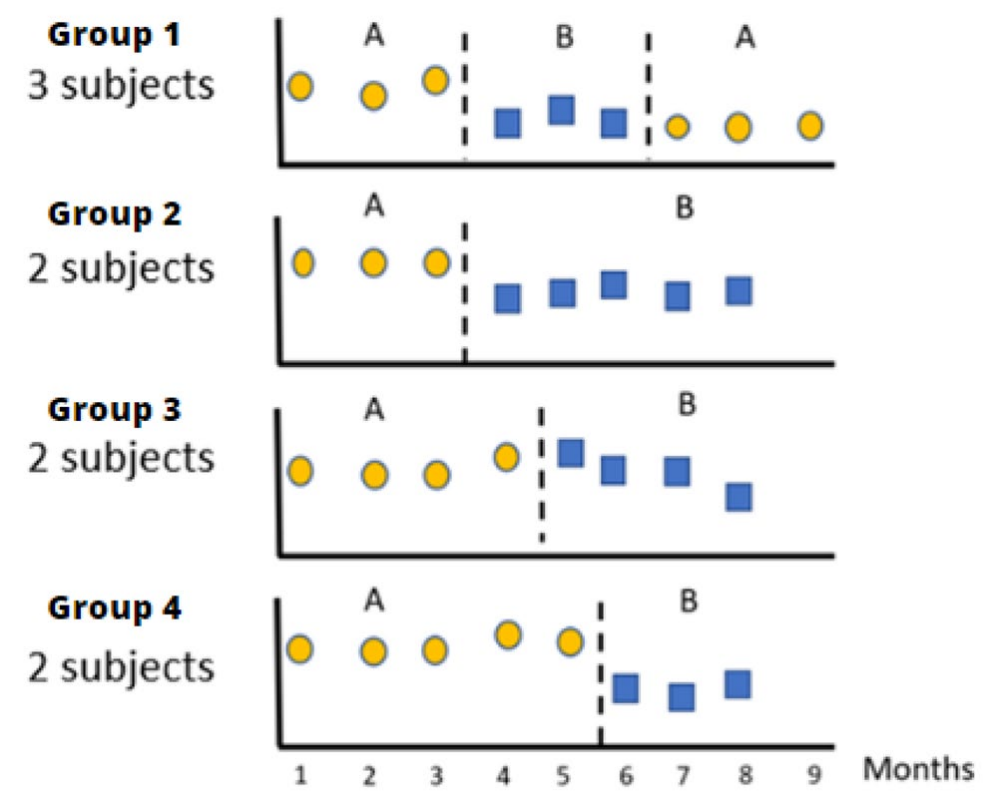
Summary of the groupings made and the different stages of evaluation. Baseline measurements are represented with circles; intervention measurements by squares; and dotted lines indicate either the beginning or end of the intervention

A mixed single-case design was chosen, with a multiple baseline design across subjects. Four groups responded to an AB scheme (A being the baseline and B the intervention), in which a new post-treatment baseline phase was added to one of the groups, which would respond to an ABA scheme (Fig. 1).

Following the current standards for these type of studies (Kratochwill et al., 2013), the design contemplates the existence of more than three participants and at least three measurement points in each phase. In addition, the allocation of each participant to each intervention group and the times at which each participant started the intervention were randomly decided by drawing lots.

In the present design the independent variable is the intervention programme, and the dependent variable is the postural control.

### Participants

A sample of nine autistic children belonging to different organisations of ASD in Castellón (Spain) was selected. The sample was recruited from volunteer parents willing to collaborate throughout the development of the intervention programme. The criteria required for inclusion in the sample were as follows: having a clinical diagnosis of ASD with a degree of affectation 1 or 2 issued by a health centre; being between 9 and 12 years oldnever having ridden a horse; and being able to respond to simple orders (Table 1). Candidates with serious behavioural problems who were taking medication that could affect the nervous system or who were overweight were excluded, the latter for animal ethics and security reasons. Eight participants were male, and one was female.

**Table 1:** Demographic data. Age of the participants

	Age	Age in months
Subject 1	Seven years and two months	86
Subject 2	Six years and two months	74
Subject 3	Six years and one month	73
Subject 4	Six years and one month	73
Subject 5	Eleven years and one month	133
Subject 6	Nine years and two months	110
Subject 7	Eleven years and five months	137
Subject 8	Eight years and four months	100
Subject 9	Eleven years and five months	137
**Mean age**		**102**
**Standard deviation**		**26**

Before the study began, the participants’ legal guardians signed an informed consent document for their children to participate in the study. Both the informed consent and the information collected in the study were in line with the recommendations of the 2013 Declaration of Helsinki. This research was approved by the Deontological Commission of the Universitat Jaume I of Castellón (Spain), with reference code CD/54/2019.

### Instruments

To assess the objective of postural control, we used a Medicapteurs branded posturographic platform, model T-Plate®, approved by the French Posturology Association. Through the pressures exerted by the subjects’ bodies on the platform sensors, the T-Plate evaluated the area covered by the centre of pressure (COP).

T-Plate offers the possibility of studying alterations to the different sensory systems involved in postural control by analysing multiple parameters such as scanning area, angle of travel, average velocity, lateral velocity, antero-posterior velocity, maximum average lateral and antero-posterior forces, and others.

In the present study we have only considered the average velocity with which the centre of pressure (AVCOP) moves, which is regarded as a parameter that correlates with the efficiency of postural control and therefore with balance (Viguier, 2009). The AVCOP is expressed in millimetres per second (mm/s). Magnitude interpretation considers that the smaller the area of deviation of the COP and, therefore, the less time spent in covering this area, the better the postural stability (Leanderson et al., 1996).

### Data Collection Procedure and Protocol

The nine participants were randomly assigned to four groups that started the intervention in consecutive months. Figure 1 shows a graph illustrating the grouping and the different moments of the evaluation.

Following the proposal of Hsu et al. (2009) postural control was evaluated under four experimental conditions: on a hard surface with participants’ eyes open; on a hard surface with participants’ eyes closed; on a padded surface with participants’ eyes open; and on a padded surface with participants’ eyes closed. This protocol enables analysis of the predominance of the sensory channel used in postural control. Closing the eyes sets in motion the proprioceptive and vestibular channels, and the padded surface inhibits proprioceptive stimuli by foregrounding the vestibular information.

For tests with closed eyes, we used a mask. For the padded surface we used a soft foam pad (50 × 50 × 6 centimetres and a density of 40 kg/m^3^).

In accordance with the work of Gagey and Weber (2004), the measurements were taken as follows: with the direct help of an adult, participants stood on the platform barefoot with their feet at an angle of 30º, their heels two centimetres apart, and their arms by their sides. Once these requirements had been met, each participant was given the following instruction: “Stand as still as possible, looking at the point in front of you.”

At the count of three the platform was activated to analyse the participant’s movements. Two measurements were taken each time, and the best of them was used.

Nine evaluations (one for every month) of each participant were performed throughout the study in order to have enough points to establish the baseline and the intervention line.

### Intervention Programme Design

The work was carried out over a nine-month period. Participants attended regularly at a rate of two 45-minute sessions per week for at least three months. Table 2 details the number of sessions that each participant received. A design comprising different length of intervention was made to observe whether the time of intervention influenced the results. Adherence to programme scheduled was 100%.

**Table 2:** Weeks of intervention per participant

	Weeks of intervention
Subject 1	11 weeks
Subject 2	11 weeks
Subject 3	11 weeks
Subject 4	22 weeks
Subject 5	22 weeks
Subject 6	18 weeks
Subject 7	18 weeks
Subject 8	14 weeks
Subject 9	14 weeks

The intervention programme for improving balance and postural control is based on the performance of several different exercises on a horse. The exercises are divided into three large blocks of increasing difficulty: with the horse standing, walking, and trotting. Within each block the exercises also increase in difficulty: holding the cinch with the eyes open and closed, and not holding the cinch with the eyes open or closed. A mask is used for the exercises with eyes closed. All these exercises have been standardised and measured with a rubric that is both a complementary evaluation system and a guide to the exercises to be followed (Vives-Vilarroig et al., 2021).

The participant mounts the horse barefoot (wearing socks) and is led by a guide and two safety monitors who remain by his/her side. No saddle or stirrups are used. The horse’s back is protected by two padded saddlebags held in place with a girth strap so that the participant can hold on.

### Data Analysis

Following the recommendations of Parker et al. (2011) and Kratochwill et al. (2013), a visual analysis of the changes in level, trend, and latency of the intervention with respect to the baseline was carried out in the first instance. To complete this analysis, two numerical indicators were provided since the visual interpretation of changes was not always evident.

For the analysis of individual differences, the NAP statistic (percentage of non-overlap between all pairs) is shown. This method is an adaptation made for single-case studies by Parker et al. (2011) of Mann Whitney’s test and shows the magnitude of the effect between adjacent phases. For their interpretation, Parker and Vannest (2009, p. 4) examine the size of the effect between phases by considering the following figures: large effect between 93% and 100%; medium effect between 66% and 92%; and finally, weak effect between 0% and 65% of the sample. In addition, we calculated Cohen’s d (1988). For its interpretation as an indicator of the magnitude of effect between adjacent phases of a design n = 1, the restrictive criteria of Harrington and Velicer (2015) should be used: small effect 0–0.99; medium effect 1–2.49 and large effect > 2.50.

For analysis of the combined effect of all participants between adjacent phases, the Between-case standardised mean difference (BC–SMD) effect magnitude indicator, known as The HPS d statistic (Hedges et al., 2013), is provided. The value of BC–SMD is interpreted as Cohen’s d (1988) since it must be understood as if it were a group study: small effect > 0.2, medium > 0.5 and grade > 0.8. The calculations were made with the web-based calculator scdhlm (Valentine et al., 2016; Pustejovsky, 2016).

## Results

The results obtained for the average velocity of the centre of pressure (AVCOP) are presented according to the four different experimental conditions studied: bipodal position on a hard surface with eyes open; bipodal position on a hard surface with eyes closed; bipodal position on a padded surface with eyes open; and bipodal position on a padded surface with eyes closed.

### Bipodal Position on a Hard Surface with Eyes Open and Closed

**Fig. 2:**
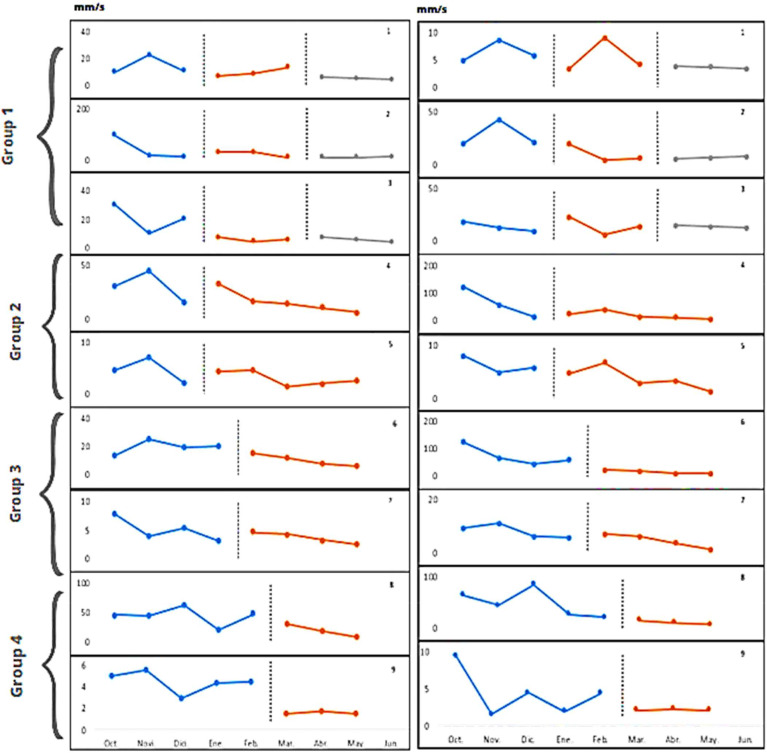
Baseline and intervention line graphs of AVCOP results (mm/s) on hard surface with participants’ eyes open (left) and closed (right). Numbers on the right side of the graph indicate the subject to which they correspond. The horizontal axis indicates the temporal scale, each point representing a consecutive month. The scale in the vertical axis corresponds to the AVCOP expressed in mm/s. Vertical dotted lines separate the baseline before the intervention (A left), the intervention (B), and the post-treatment baseline (A right, when measured). Grouping in the left side correspond to those indicated in Fig. 1

Visual analysis of the graphs (Fig. 2) reveals no evident pattern of immediate level change in most participants, although a change in the trend of the intervention line is intuited, the effect being appreciated from the second and third month of intervention.

Moreover, analysis of the reversal lines of the first three cases shows that the effect of the intervention was maintained three months after the end of the intervention programme (Tables 3 and 4).

**Table 3:** Descriptive and contrasting statistics of AVCOP between baseline and intervention line on a hard surface

*Subject*	*Number of weeks of Intervention*	*Eyes*	$\bar x\, BL$	$\bar x\, Int$	*NAP*	*Z*	*P p*	*d*
1	11	Open	14.00	7.13	0.77	-1.091	0.275	0.995
		Closed	6.36	4.59	0.66	-0.654	0.512	0.555
2	11	Open	41.40	16.90	1	-1.964	0.049*	2.683
		Closed	28.03	8.30	1	-1.964	0.049*	2.683
3	11	Open	20.20	5.65	1	-1.964	0.049*	2.683
		Closed	13.50	14.01	0.44	-0.218	0.495	0.197
4	22	Open	30.20	15.76	0.80	-1.341	0.179	1.078
		Closed	64.73	18.64	1	-2.236	0.025*	2.582
5	22	Open	4.50	3.02	0.80	-1.341	0.179	1.078
		Closed	6.23	3.80	0.86	-1.414	0.157	1.155
6	18	Open	19.50	10.32	0.93	-2.020	0.042*	2.043
		Closed	72.72	15.87	1	-2.309	0.020*	2.828
7	18	Open	5.00	3.60	0.68	-0.866	0.386	0.643
		Closed	8.02	4.35	0.81	-1.154	0.248	1.187
8	14	Open	43.50	17.86	0.93	-1.937	0.052	1.881
		Closed	47.54	10.40	1	-1.964	0.033*	2.683
9	14	Open	4.42	1.56	1	-2.236	0.020*	2.582
		Closed	4.30	2.13	0.60	-0.447	0.654	0.320

Note: $\bar x\, BL$: Mean baseline (mm/s); $\bar x\, Int$: Mean intervention line (mm/s); *NAP*: Non-overlap of all baseline and treatment phase pairs. It measures the percentage of data that improve from the baseline (Parker & Vannest, 2009). Significant values (p < 0.05) have been highlighted in bold and marked with an asterisk. Cohen’s d is interpreted according to Harrington and Velicer’s (2015) criteria: small effect 0–0.99; medium effect 1–2.49; large effect > 2.50

**Table 4:** Standardised mean difference between cases (BC–SMD) on a hard surface

*Eyes*	$\bar x\, BL$	$\bar x\, Int$	*BC–SMD*	*L. Inf. 95%*	*L. Sup. 95%*
Open	20.30	9.08	0.703	0.273	1.162
Closed	27.93	9.12	0.526	0.018	1.104

Note: $\bar x\, BL$ is the baseline average of all the participants (mm/s); $\bar x\, Int$ is the intervention line average of all the participants (mm/s); *BC–SMD* analyses the difference between standardised means between cases (Pustejovsky, 2016) and is interpreted with Cohen’s d: small effect 0.2–0.49, medium effect 0.5–0.79, large effect > 0.8. *L. Inf*: Lower bound; *L Sup*.: Upper limit

### Bipodal Position on a Padded Surface with Participants’ Eyes Open and Closed

**Fig. 3:**
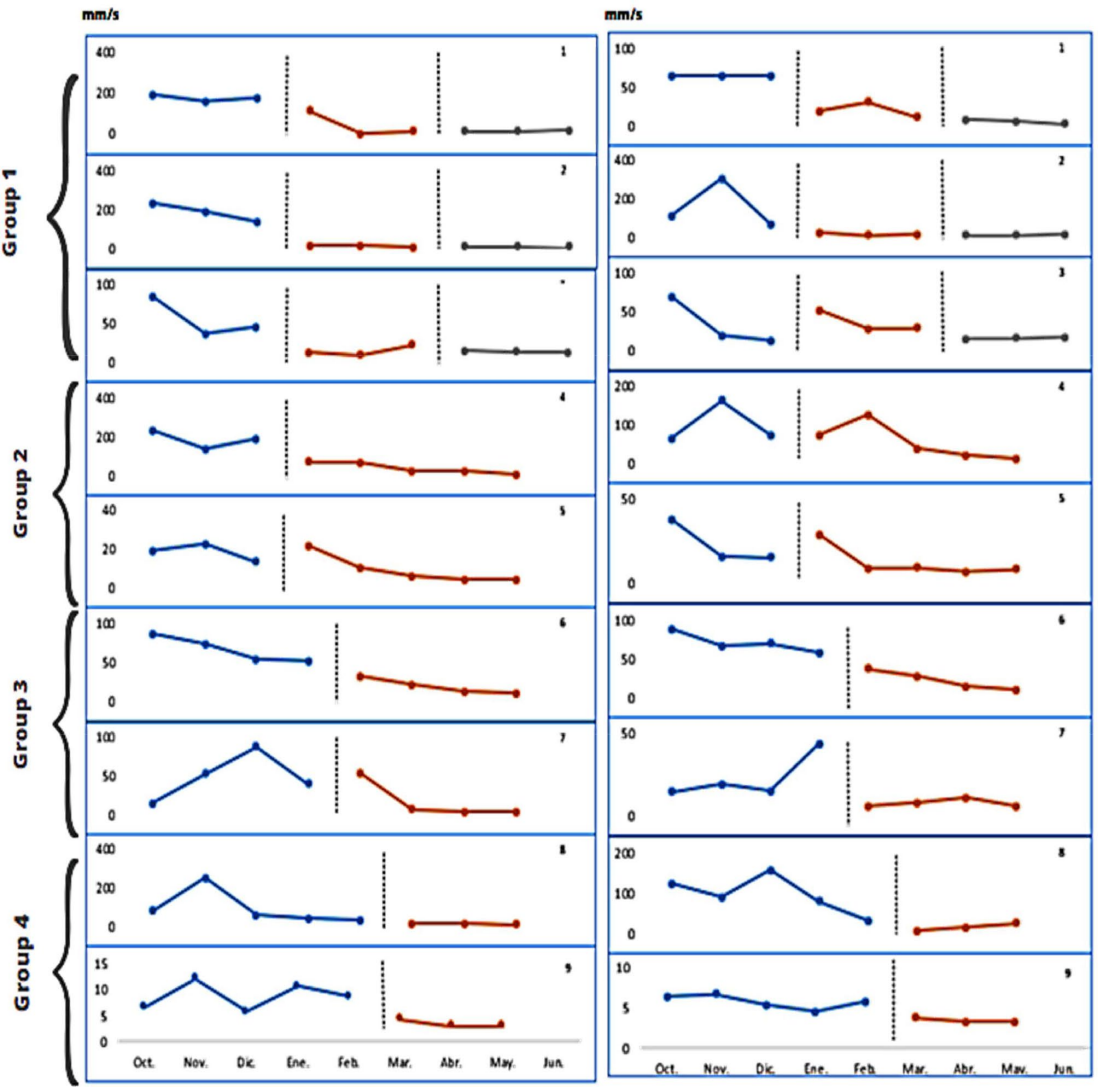
Baseline and intervention line graphs of AVCOP results (mm/s) on a padded surface with participants’ eyes open (left) and closed (right). Numbers on the right side of the graph indicate the subject to which they correspond. The horizontal axis indicates the temporal scale, each point representing a consecutive month. The scale in the vertical axis corresponds to the AVCOP expressed in mm/s. Vertical dotted lines separate the baseline before the intervention (A left), the intervention (B), and the post-treatment baseline (A right, when measured). Grouping in the left side correspond to those indicated in Fig. 1

Also, visual analysis of the graphs (Fig. 3) reveals no evident pattern of immediate level change in most participants. However, a change in the trend of the intervention line is observed, the effect being seen from the second and third month of intervention. Moreover, analysis of the reversal lines of the first three participants shows that the effect of the intervention programme is maintained after three months.

The quantitative data indicate that in eight of the nine participants the effect of the intervention programme was large (Table 5). In addition, analysis of the aggregate data (Table 6) indicates that the magnitude of the effect of the intervention programme is large for both open-eye (BC–SMD = 1.033) and closed-eye (BC–SMD = 0.838) conditions.

**Table 5:** Descriptive and contraction statistics of AVCOP between baseline and intervention line on a padded surface

*Subject*	*Number of weeks of Intervention*	*Eyes*	$\bar x\, BL$	$\bar x\, Int$	*NAP*	*Z*	*p*	*d*
1	11	Open	177.90	28.96	1	-1.964	0.049*	2.683
		Closed	61.13	12.83	1	-1.9640	0.049*	2.683
2	11	Open	182.50	11.73	1	-1.964	0.049*	2.683
		Closed	164.96	16.35	1	-1.964	0.049*	2.683
3	11	Open	56.20	14.85	1	-1.964	0.049*	2.683
		Closed	34.43	26.66	0.33	− 0.654	0.512	0.555
4	22	Open	186.50	37.32	1	-2.236	0.025*	2.582
		Closed	102.46	55.42	0.80	-1.5492	0.121	1.309
5	22	Open	18.63	9.48	0.86	-1.639	0.101	1.423
		Closed	23.16	12.24	0.86	-1.639	0.101	1.423
6	18	Open	65.95	18.82	1	-2.121	0.033*	2.267
		Closed	70.67	23.42	1	-2.309	0.020*	2.828
7	18	Open	49.55	17.95	0.81	-1.443	0.148	1.187
		Closed	23.35	7.75	1	-2.309	0.020*	2.828
8	14	Open	96.50	18.23	1	-2.121	0.033*	2.267
		Closed	96.54	16.33	1	-2.121	0.033*	2.267
9	14	Open	8.76	3.36	1	-2.236	0.025*	2.582
		Closed	5.72	3.43	1	-2.236	0.025*	2.582

Note: $\bar x\, BL$: Mean baseline (mm/s); $\bar x\, Int$: Mean intervention line (mm/s); *NAP*: Non-overlap of all baseline and treatment phase pairs. It measures the percentage of data that improve from the baseline (Parker & Vannest, 2009). Significant values (p < 0.05) have been highlighted in bold and marked with an asterisk. Cohen’s d is interpreted according to Harrington and Velicer’s (2015) criteria: small effect 0–0.99; medium effect 1–2.49; large effect > 2.50

**Table 6:** Standardised mean difference between cases (BC–SMD) on a padded surface

*Eyes*	$\bar x\, BL$	$\bar x\, Int$	*BC–SMD*	*L. Inf. 95%*	*L. Sup. 95%*
Open	93.61	17.85	1.033	0.409	1.717
Closed	64.71	19.37	0.838	0.270	1.431

Note: $\bar x\, BL$ is the baseline average of all the participants(mm/s); $\bar x\, Int$ is the intervention line average of all the participants (mm/s); *BC–SMD* analyses the difference between standardised means between cases (Pustejovsky, 2016) and is interpreted with Cohen’s d: small effect 0.2–0.49, medium effect 0.5–0.79, large effect > 0.8. *L. Inf*: Lower bound; *L Sup*.: Upper limit

## Discussion

The objective of this paper was to study whether a programme of therapeutic horse-assisted activities can improve postural control in autistic children. For this purpose, an experimental design of a multiple base group between subjects was carried out, with four groups responding to an AB scheme (A being the baseline and B the intervention). A new reversal phase (ABA) was added to one of the groups to obtain data about maintaining the treatment effect in the absence of intervention.

The baseline data on the two surfaces studied show that the starting point is far removed from the regulatory data for the corresponding age in neurotypical children. Our group of participants’ AVCOP on a hard surface was 20.3 mm/s for open eyes and 27.9 mm/s for closed eyes, the normative scores for both conditions being around 3.5 mm/s. Our group’s AVCOP on a padded surface was 93.6 mm/s (open eyes) and 64.7 mm/s (closed eyes), the standard scores also being around 3.5 mm/s (Shams et al., 2020).

The fact that the worst scores were obtained on a padded surface (inhibition of proprioceptive stimuli) and with the participants’ eyes open (93.7 mm/s) seems to indicate that vestibular input and poor use of visual information confer a peculiar style of postural control to autistic children (Doumas et al., 2016). Regarding this issue, some characteristics have been observed in this research corroborating the scientific literature, such as the specific deficit in the use of vision for action. Some authors have highlighted this fact, trying to explain that, besides limitations in the reception and processing of vestibular stimuli, there is a specific deficit in the use of vision for action in autistic children, which can explain in part the social and motor difficulties associated to this syndrome (Morris et al., 2015).

Another feature observed is postural impairment, which in the case of people with ASD may be related to abnormal neurophysiology based on atypical connectivity between brain regions during sensory stimulation (Lim et al., 2017), as they have structural or functional impairments (Allen & Courchesne, 2003; Nayate et al., 2005). These impairments are exacerbated in situations where children with ASD experience a disruption of some of the channels that support sensory integration, as corroborated by previous studies indicating that postural flopping (lack of postural control) is in part due to poor integration of vestibular, somatosensory and visual inputs (Kohen-Raz et al., 1992; Molloy et al., 2003). This atypical connectivity would determine the poor processing of sensory signals received by some sensory channels and the multiple sensory processing of these signals (Lim et al., 2017).

Regarding the effects of the intervention programme, the results indicate that the intervention with horses had a positive effect on the postural control of participants with autism. This effect was moderate when participants were evaluated on hard surfaces, but large when they were evaluated on padded surfaces, even when their eyes were closed.

It should be noted that the greatest effect occurred when data were obtained on padded surfaces with participants’ eyes open, which seems to indicate that working with horses produces sensory stimulation in all channels, but especially in vestibular and visual stimuli. This fact is related to the observations made by some authors (Bronson et al., 2010; Muñoz Lasa et al., 2015; Tseng et al., 2013; Olivier et al., 2017) who consider that riding a horse involves intensive exercise which is highly specific for improving balance, since the horse’s gait induces continuous changes in the rider’s centre of gravity requiring positional readjustments as the horse moves.

This continuous repositioning due to the movements of horse-riding has been described as being facilitated by the vestibular system (Campbell & Garrett, 1996). It is perhaps the greatest success of this intervention, since the vestibular stimulation that involves riding a horse is difficult to simulate elsewhere. Obviously, this vestibular stimulation requires of a special and specific training.

However, despite the statistically significant effect of the intervention programme on participants’ postural control, the results at the end of the intervention programme were not within the normotypical values for the corresponding age. This fact may be related to the duration and frequency of the intervention programme, but also to the persistence of motor symptoms in autism, as they are usually still present even in the adult population (Lim et al., 2017). Nevertheless, the persistence of symptoms encourages us to think about planning longer intervention programmes than those followed in our work. In this study, no significant differences were observed between the number of sessions assigned to each group in the range studied, due to the limited size number and the high dispersion of data associated to ASD individuals.

Although there is very little information about the therapeutic and re-educational alternatives to improve the postural control of people with autism, some experimental programmes have offered encouraging results (Cheldavi et al., 2014).

These programmes have been successful to a greater or lesser extent. However, future research should provide information about which programme is most effective for different conditions and age groups, and about the relevance, feasibility, and cost effectiveness of the different techniques. In addition, the intensity and duration of the sessions and the permanence in time of the achievements in the medium and long term should be investigated.

Finally, we would like to point out that care of postural control in autistic children in whom this ability is affected is an indispensable task since this deficit directly influences personal autonomy (Fears et al., 2022) and the successful performance of routine activities (Travers et al., 2017). On the other hand, the fact that deficits in postural control can be observed before the appearance of social and communicative symptoms (Radonovich et al., 2013) allows us to venture that appropriate early intervention may decisively influence the severity of the evolution of the disorder (Nayate et al., 2005). In this regard we support the suggestions of Li et al. (2021) who emphasise that early intervention in children with ASD is very important for improving postural control through the development of postural stability and motor function. These authors suggest that interventions could include opportunities to experience different body movements or positions that require different postural control strategies. In addition, several authors (Muñoz Lasa et al., 2015) argue that much of the ability to learn comes from the ability to integrate sensory information in the first years of life. However, they conclude that more research is needed on deficits in postural control complexity for young autistic children, along with evaluation of the efficacy of early interventions in this aspect.

Conclusively, the data here presented reveal that the horseback-riding intervention programme significantly improved participants’ postural control. Some factors that might explain this improvement could be that they were developed in a highly structured and motivating context providing intense and specific multisensory stimulation adjusted to the specific needs of the participants.

### Limitations

Once the effect of the intervention programme on the postural control of autistic children has been demonstrated with multiple baseline across subjects design, efforts should be made to carry out group, randomised, and double-blind experimental designs with adequate statistical power.

The limited sample used does not allow investigation of the effect of the treatment programme on some differential characteristics such as age, gender, or the level of severity of the disorder.

Regarding our design in particular, we understand that there is appreciable variability in the baseline of some of the participants, although participants with baselines with more than three evaluations have tended towards stabilisation. Presumably, a longer baseline period would have enabled a clearer visual analysis of the effect of the intervention.

On the other hand, although a reversionary design (ABA) with three measurements after the intervention programme was carried out in one of the groups, we have no information about the long-term effects of the intervention programme. It was not possible to obtain comparative results between the groups to which different intervention times were applied, in part due to the heterogeneity of each subject with ASD and their own evolutionary development.

Finally, it should be noted that, although the participants were selected on the basis of their ability to follow orders, their difficulties in attention and concentration, combined with the high sensitivity of the posturography sensors, meant that the measurements were not free of difficulty, which also resulted in higher data dispersion.

These limitations are the basis for further research.
